# The gut microbial metabolite indole-3-aldehyde alleviates impaired intestinal development by promoting intestinal stem cell expansion in weaned piglets

**DOI:** 10.1186/s40104-024-01111-7

**Published:** 2024-11-08

**Authors:** Jiaqi Zhang, Yahui Chen, Xin Guo, Xuan Li, Ruofan Zhang, Mengting Wang, Weiyun Zhu, Kaifan Yu

**Affiliations:** 1 Laboratory of Gastrointestinal Microbiology, Jiangsu Key Laboratory of Gastrointestinal Nutrition and Animal Health, College of Animal Science and Technology, Nanjing Agricultural University, Nanjing, 210095 China; 2 National Center for International Research on Animal Gut Nutrition, Nanjing Agricultural University, Nanjing, 210095 China; 3 Wujiang Animal Health Inspection Institute, Suzhou, 215200 China

**Keywords:** Indole-3-aldehyde, Intestinal development, Intestinal microbiota, Intestinal stem cell, Weaned piglets

## Abstract

**Background:**

Weaning stress-induced diarrhea is widely recognized as being associated with gut microbiota dysbiosis. However, it has been challenging to clarify which specific intestinal microbiota and their metabolites play a crucial role in the antidiarrhea process of weaned piglets.

**Results:**

In this study, we first observed that piglets with diarrhea exhibited a lower average daily gain and higher diarrhea score, and elevated levels of lipopolysaccharide (LPS) and D-lactate (D-LA) compared to healthy piglets. Subsequently, we analyzed the differences in intestinal microbial composition and metabolite levels between healthy and diarrheal weaned piglets. Diarrheal piglets demonstrated intestinal microbiota dysbiosis, characterized primarily by a higher Firmicutes to Bacteroidota ratio, a deficiency of *Lactobacillus amylovorus* and *Lactobacillus reuteri*, and an increased abundance of *Bacteroides sp.HF-5287* and *Bacteroides thetaiotaomicron*. Functional profiling of the gut microbiota based on Kyoto Encyclopedia of Genes and Genomes (KEGG) data was performed, and the results showed that tryptophan metabolism was the most significantly inhibited pathway in piglets with diarrhea. Most tryptophan metabolites were detected at lower concentrations in diarrheal piglets than in healthy piglets. Furthermore, we explored the effects of dietary indole-3-aldehyde (IAld), a key tryptophan metabolite, on intestinal development and gut barrier function in weaned piglets. Supplementation with 100 mg/kg IAld in the diet increased the small intestine index and improved intestinal barrier function by promoting intestinal stem cell (ISC) expansion in piglets. The promotion of ISC expansion by IAld was also confirmed in porcine intestinal organoids.

**Conclusions:**

These findings revealed that intestinal microbial tryptophan metabolite IAld alleviates impaired intestinal development by promoting ISC expansion in weaned piglets.

## Introduction

Weaning presents a significant challenge for intestinal development in piglets, as they must adapt to rapid changes in diet, physical environment, and social interactions. Perturbations during this critical window can have long-term effects on phenotype and intestinal function [1]. Piglets suffering from weaning stress typically exhibit decreased feed intake, followed by disruption of gut microbiota, digestion and absorption capacity, and intestinal barrier function, eventually leading to diarrhea, and restricted growth [2–4]. In modern pig production, early weaning is often employed to increase sow productivity, which in turn increases the susceptibility of piglets to weaning stress. Therefore, it is significant to study how to improve the intestinal health of piglets during weaning.

Weaning is a critical window for the development of intestinal barrier function [5]. Numerous studies have reported that weaning stress is associated with dysfunction of the intestinal barrier, including morphological damage and breaches in the inner mucus layer [1, 6]. Moreover, early weaning has been shown to affect the proliferation, differentiation, and shedding of intestinal epithelial cells [7]. These adverse effects on intestinal morphology and function often lead to post-weaning diarrhea and other intestinal diseases [5]. Therefore, exploring strategies to improve intestinal development in weaned piglets is of great significance.

The intestinal tract of piglets houses a diverse and complex microbial community, which profoundly influences the development of the intestinal epithelium, the maintenance of the intestinal barrier, and the functioning of the immune system [8, 9]. During the weaning period, there are significant quantitative and qualitative changes in the composition of the intestinal microbiota of piglets [10, 11]. Notably, weaning stress is often accompanied by disturbances in gut microbial diversity and can lead to post-weaning diarrhea [12]. The detrimental effects of diarrhea-induced gut microbiota dysbiosis on host health have been well documented. Additionally, growing evidence suggests that metabolites produced by gut microbiota play a vital role in host health by regulating intestinal development and nutrient metabolism [13, 14]. However, it has been challenging to clarify the specific intestinal microbiota and their metabolites that play a crucial role in mitigating diarrhea in weaned piglets.

Recently, increasing attention has been focused on gut microbiota-derived tryptophan catabolites due to their positive impact on host physiology [15, 16]. Several studies have shown that gut microbial species such as *Escherichia coli*, *Clostridium* spp., *Peptostreptococcus* spp., *Lactobacillus* spp., and *Bacteroides* spp., convert tryptophan into various metabolites, including indolelactic acid, indoleacrylic acid, indolepropionic acid, indolealdehyde, and tryptamine, through different metabolic pathways. These tryptophan catabolites can influence host health by activating the immune system, enhancing the intestinal epithelial barrier, exerting antioxidative effects in the systemic circulation, and regulating gut microbial composition [17–21]. However, the role of these tryptophan catabolites in regulating intestinal homeostasis during the weaning process in piglets remains unclear.

Here, we first assessed the intestinal barrier function in both healthy and diarrheal weaned piglets. Second, intestinal microbial composition and microbial metabolite levels were analyzed using 16S rRNA sequencing, metagenomics, and targeted metabolomics. Furthermore, we explored the effects of dietary indole-3-aldehyde (IAld), a microbial key tryptophan metabolite, on intestinal development and barrier function in weaned piglets. Finally, we elucidated the mechanism by which IAld affects intestinal development using porcine intestinal organoids.

## Materials and methods

### Experiments for the selection of healthy and diarrheal piglets

In this study, fifty crossbred male piglets (Duroc × Landrace × Yorkshire) were weaned at 21 days of age and fed for one week. The diet provided to the piglets adhered to the National Research Council (NRC, USA) 2012 [22] nutrient requirements. All piglets had free access to milk/feed and water according to standard feeding management practices, with no antibiotics or immunological measures added. The health and fecal status of the piglets were closely monitored during the first week after weaning to assess the severity of diarrhea. The diarrhea score was recorded as follows: 0 = normal, firm feces; 1 = soft feces, possibly slight diarrhea; 2 = unformed, moderately fluid feces; and 3 = very watery and frothy diarrhea [23]. Piglets with a score of 0 or 1 for at least 3 continuous days and no other diseases were designated as healthy piglets, while those piglets with a score of 3 for at least 3 continuous days were classified as diarrheal piglets. Ten healthy piglets and ten diarrheal piglets were selected to provide fecal samples for further analysis. The body weight at 21 d (initial weight) and 28 d (final weight) was recorded to calculate the average daily gain of the piglets. Fresh fecal samples were collected for 16S rRNA gene sequencing, metagenomic sequencing, and metabolomic analysis. Blood samples were collected to obtain serum for further analysis.

### Serum biochemical indicator analysis

The concentration of lipopolysaccharide (LPS) in the serum was determined using a commercial ELISA kit (Jiangsu Meimian Industrial Co., Ltd., China). The serum D-lactate (D-LA) concentration was determined using an assay kit (Jiangsu Meimian Industrial Co., Ltd., China).

### 16S rRNA gene sequencing analysis

Total bacterial genomic DNA was extracted from the fecal samples of healthy and diarrheal piglets using a DNA isolation kit (Qiagen, Hilden, Germany). 16S rRNA gene sequencing was performed to study the differences in the fecal microbiota between the healthy and diarrheal piglets, focusing on microbial diversity and community composition at the phylum and genus levels. The V3–V4 region of the 16S rRNA gene was amplified using universal primers 341F (5′-CCTAYGGGRBGCASCAG-3′) and 806R (5′-GGACTACNNGGGTATCTAAT-3′). The amplified products were sequenced on the Illumina MiSeq platform. High-quality reads were obtained by quality control and then were imported into QIIME2 for analysis. Principal coordinate analysis (PCoA) based on unweighted UniFrac distances was used to analyze all OTUs via OmicStudio tool at https://www.omicstudio.cn/tool. Differences in microbial results between the two groups were calculated by the nonparametric test with *P* < 0.05.

### Metagenomic sequencing analysis

Metagenomic sequencing was performed to compare the microbial community composition at the species level and microbial function in the fecal microbiota of healthy (*n* = 10) and diarrheal piglets (*n* = 10). Sequencing was performed on the MGISEQ 2000 platform (PE150). High-quality reads were obtained after filtering adapters, low-quality reads, and host genomic DNA contamination from the raw sequencing data by the SOAPnuke software and Bowtie2 package. Subsequently, MEGAHIT was used to assemble high-quality reads from each sample. After this, MetaGeneMark was used to predict the contigs from each sample, and the reads clustered into a nonredundant data set by CD-HIT. Then, the nonredundant reads were mapped against the protein sequence of the Kyoto Encyclopedia of Genes and Genomes (KEGG) database using DIAMOND software. The abundance of bacteria at the species level was analyzed. The differences in species and pathways between healthy piglets and diarrheal piglets were calculated using a nonparametric test. The relative abundance of genes involved in the KEGG was calculated. The potential KEGG Orthologys (KOs) annotated to tryptophan metabolism (map00380) were also analyzed. Based on the information available for the genes encoding these KOs, their origin at the genus level was determined, and their relative abundance was calculated using the transcripts per kilobase million (TPM).

### Tryptophan metabolite quantification

Targeted metabolomics was performed to determine the concentrations of tryptophan catabolites in the fecal samples of healthy and diarrheal pigs. Briefly, 10 mg of lyophilized fecal sample was suspended in 140 µL of 50% methanol/water solution. Following homogenization, the mixture was centrifuged at 12,000 r/min for 10 min at 4 °C. The supernatant and standard compounds of the target tryptophan metabolites were then subjected to a derivatization reaction. The chemical derivatization reaction included 5-dimethylamino-naphthalene-1-sulfonyl chloride (Dns-Cl) derivatization and 5-Dimethylamino-naphthalene-1-sulfonyl piperazine (Dns-PP) derivatization, which was performed according to the method reported by Tan et al. [24]. Metabolites containing amino/phenol group were reacted with Dns-Cl, and metabolites containing carboxyl group were labeled by Dns-PP. After dilution, the mixture was centrifuged again at 4 °C and 12,000 r/min for 10 min. The resulting supernatant was analyzed for tryptophan catabolite quantification using liquid chromatography-tandem mass spectrometry (LC-MS/MS) analysis on an LC-MS QTRAP 6500+ (SCIEX). Finally, quality-control checks and data preprocessing were performed to identify and quantify the target metabolites.

### Indole-3-aldehyde treatment experiments in piglets

Sixteen male weaned Landrace piglets, 28 days of age, with similar weight (9.06 ± 0.28 kg) were randomly divided into two groups. The piglets were fed with a basal diet, or the basal diet supplemented with 100 mg/kg IAld (catalog number: 129445, Sigma-Aldrich, St. Louis, MO, USA) for 14 d. All the piglets had free access to feed and water according to the standard feeding management practices without any antibiotics added or immunological measures. At the end of the feeding trial, the small intestinal sections including jejunum and ileum samples (5–6 cm in the middle segment) were collected. The small intestine index was calculated using the formula: small intestine index (%) = small intestine weight (g)/body weight (g) × 100. Some tissues were fixed in 4% paraformaldehyde for subsequent histological and immunofluorescence staining. Other samples were stored at −80 °C for further analysis.

### Histological analysis and immunofluorescence staining

Small intestinal sections (1–2 cm in the middle segment) were fixed in 4% paraformaldehyde, dehydrated with alcohol, embedded in paraffin blocks, and sliced into 5 μm sections. These sections were stained with H&E for histological and immunofluorescence analysis. All H&E-stained images were obtained using an Eclipse 80i microscope (Nikon, Tokyo, Japan). The villus area was calculated following the method described in Bai et al. [25]. For immunofluorescence staining, antibodies against Olfm4, Keratin 20 (KRT20), and Villin were obtained from Cell Signaling Technology (Beverly, USA), and the antibody against Mucin2 was sourced from ABclonal (Wuhan, China). Staining for Olfm4, KRT20, Villin, and Mucin2 (ABclonal) was performed on the sections. All immunofluorescence images were captured using an inverted fluorescence microscope (Ti2-U, Nikon, Tokyo, Japan).

### RNA preparation and quantitative real-time PCR analysis

Total RNA was isolated from tissue or organoids using the RNApure Total RNA kit (Aidlab, Beijing, China) according to the manufacturer’s instructions. Reverse transcription and Real-time PCR assays were performed using the PrimerScript™ reagent kit and ChamQ™ SYBR Green Real-time PCR Master Mix kit (TaKaRa Biotechnology Co. Ltd., Dalian, China), respectively. The specific primers used are listed in Table 1.

**Table 1:** Primers used for quantitative real-time PCR

Genes	Forward sequence (5′→3′)	Reverse sequence (5′→3′)
*GAPDH*	GGTCGGAGTGAACGGATTT	CATTTGATGTTGGCGGGAT
*Muc2*	ACGGACCGCAGGGACAACG	GTGATGCTGGAGACGGAGGAGATG
*Olfm4*	CACCAGGAACATTGCCAGAG	TCTTGCCAGCCAACATTAGC
*Lgr5*	GCTGGCTGCCGTGGATGC	AGCAGGCGCAGAGGACAAG
*PCNA*	TACGCTAAGGGCAGAAGATAATG	CTGAGATCTCGGCATATACGTG
*LYZ*	AACTGCTTTGGGTGTCTTGC	GGTCTATGATCGGTGCGAGT
*AHR*	CAGCCATGGTGACTCCTCAG	ATTGCTGGGCTGTACTGCAT
*CYP1A1*	AGACCTCCTCCTCGCACTTCT	GTGGCTTCCCTCACGATTCA
*β-catenin*	GAGACGGAGGAAGGTCCGAG	AGCTTGGGTAGCCATTGTCC
*c-Myc*	CTCGGACTCTCTGCTCTCCT	TTGTTCTTCCTCAGAGTCGCT
*Cyclin D1*	GCCCTCCGTGTCCTACTTCA	AGACCTCCTCCTCGCACTTCT

### Total protein isolation and Western blotting

The tissues and organoids were lysed with RIPI buffer (Beyotime, Shanghai, China) containing 1% PMSF (Beyotime, Shanghai, China). Equal amounts of sample lysates and a color prestained protein marker (ABclonal, Wuhan, China) were separated by sodium dodecyl sulfate/polyacrylamide gel electrophoresis (SDS/PAGE) gels and transferred to polyvinylidene fluoride membranes (PVDF) membranes. After blocking, the membranes were incubated with primary antibodies against β-actin, Lgr5, Proliferating cell nuclear antigen (PCNA), Occludin, Claudin1, β-catenin, and active β-catenin. Antibodies against β-actin, PCNA, Occludin, and Claudin1 were purchased from Proteintech Group Inc. (Wuhan, China). The anti-Lgr5 antibody was purchased from ABclonal (Wuhan, China), while antibodies against β-catenin and active β-catenin were from Cell Signaling Technology (Beverly, USA). The membranes were then incubated with secondary antibodies (ABclonal). Proteins were visualized using an enhanced ECL chemiluminescent substrate kit (Yeasen) with a FluorChem M imaging system (ProteinSimple, Inc., Santa Clara, CA, USA).

### Intestinal crypt isolation and culture

The small intestinal crypts of the pigs were isolated and cultured following the method described by Zhou et al. [26]. Briefly, the jejunal tissue from 7-day-old piglets was placed in ice-cold Dulbecco’s phosphate-buffered saline (DPBS). After removing the mesentery and villi, the jejunal tissue was cut into 5 mm × 5 mm pieces and repeatedly washed with DPBS until the supernatant was clear. The tissue pieces were then incubated in ice-cold DPBS containing 30 mmol/L ethylenediaminetetraacetic acid disodium salt (EDTA-2Na) for 30 min at 80 r/min. Crypts were collected after removing the supernatant. The crypts were resuspended in a mixture of IntestiCult organoid growth medium (Stemcell, Canada) and Matrigel (BD Biosciences, San Jose, CA, USA). The crypts were then embedded in a mixture of medium and Matrigel in a 24-well plate. After the Matrigel solidified, 500 µL of IntestiCult organoid growth medium was added to each well. The IAld group was treated with 15 µmol/L IAld. All organoids were cultured at 37 °C with 5% CO_2_. Organoid forming efficiency was determined as the proportion of the number of organoids to the number of crypts seeded, and organoid budding efficiency was calculated as the proportion of the number of budding organoids to the total number of organoids.

### Statistical analysis

The data were analyzed using the Student’s *t*-test for comparisons between two groups or one-way analysis of variance (ANOVA) followed by a post hoc Tukey test for multiple group comparisons in SPSS software version 20.0 (SPSS Inc., Chicago, IL, USA). All data are expressed as the mean ± SEM. Statistical significance was defined as *P* < 0.05 for all experiments.

## Results

### Diarrheal piglets exhibit increased serum LPS and D-LA

**Fig. 1:**
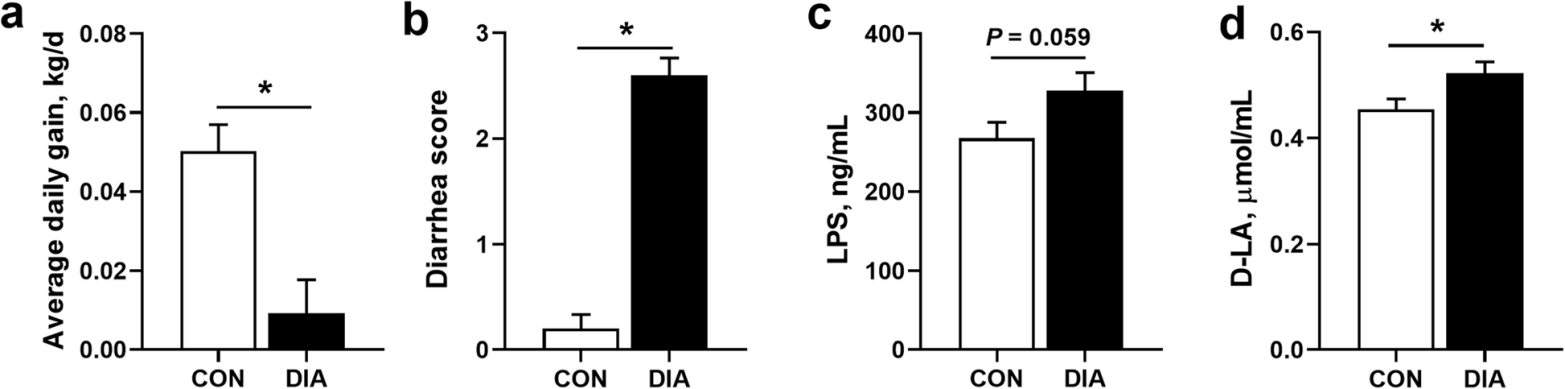
Evaluation of growth, diarrhea severity, and intestinal barrier function in healthy and diarrheal weaned piglets. **a** Average daily gain. **b** Diarrhea score. **c** The concentration of LPS in serum. **d** The concentration of D-LA in serum. Data are expressed as the mean ± SEM (*n* = 10). ^*^P < 0.05

Typical characteristics of healthy and diarrheal piglets are shown in Fig. 1. Diarrheal piglets demonstrated lower average daily gain and higher diarrhea scores compared to healthy piglets (*P* < 0.05, Fig. 1a and b). To investigate the effect of weaning diarrhea on the intestinal barrier of piglets, we measured serum levels of LPS and D-LA. The results showed that diarrheal piglets had significantly higher serum D-LA levels (*P* < 0.05) and a trend toward increased serum LPS levels (0.05 < *P* < 0.10) compared to healthy piglets (Fig. 1c and d). These findings indicated that piglets with diarrhea were most likely exhibited intestinal barrier damage.

### Diarrheal piglets exhibit gut microbial dysbiosis

**Fig. 2:**
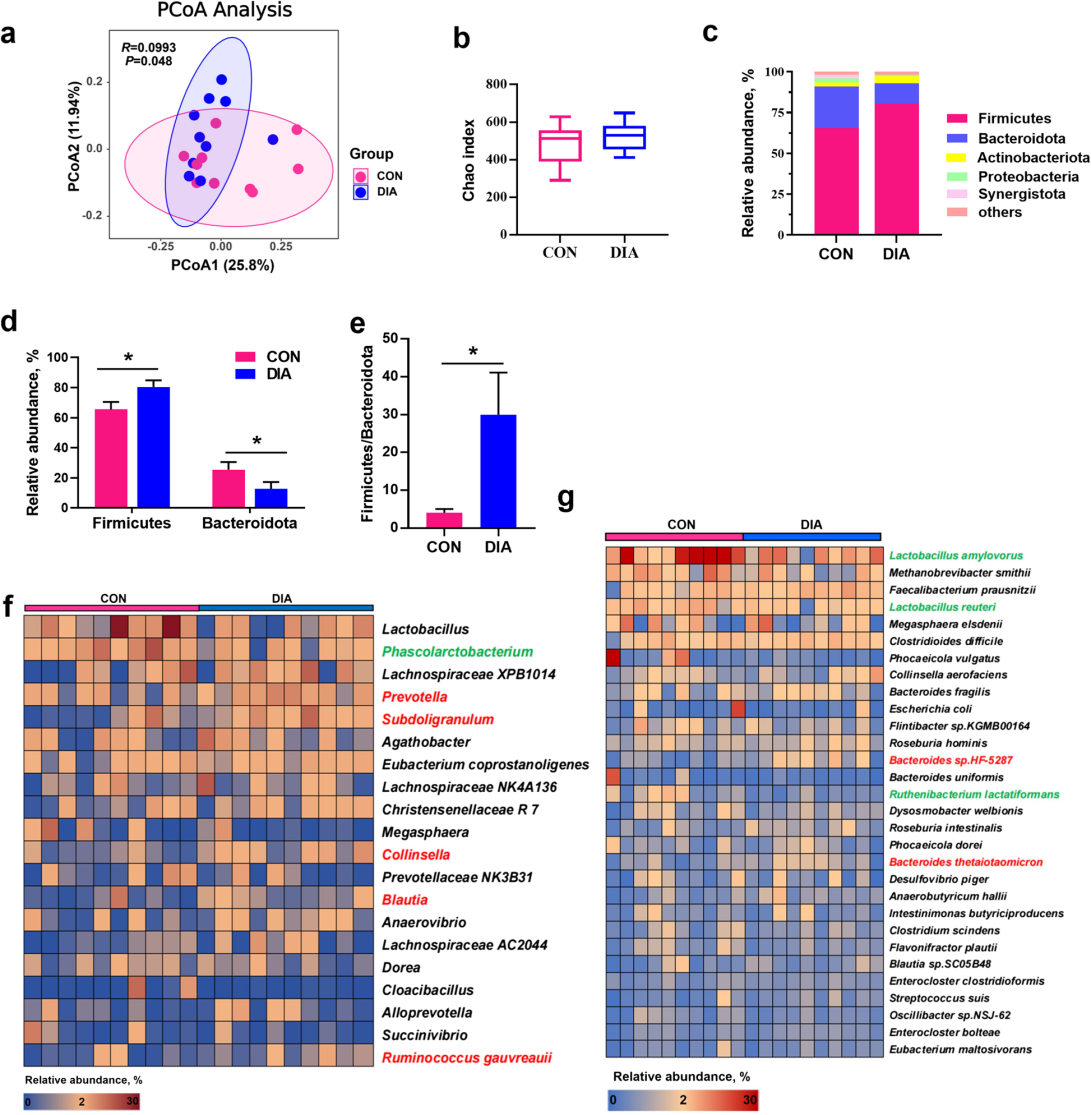
The structure and composition of fecal microbiota in healthy and diarrheal weaned piglets. **a** Principal coordinate analysis (PCoA). **b** Chao index represents the α-diversity. **c** Phylum-level distribution. **d** Relative abundance of the phyla Firmicutes and Bacteroidetes. **e** The ratio of the relative abundance of Firmicutes to Bacteroidota. **f** The heatmap visualization of the relative abundance of the top 20 genera. **g** The heatmap visualization of the relative abundance of the top 30 species. The red font represents a significant increase in the relative abundance of the bacterial genus and species compared with the CON group. The green font represents a significant decrease in the relative abundance of the bacterial genus and species compared with the CON group. Data are expressed as the mean ± SEM (*n* = 10). ^*^P < 0.05

To explore the differences in fecal microbiota between diarrheal and healthy piglets, 16S rRNA gene and metagenomic sequencing were performed to analyze the composition and functional profiles of the fecal microbiome. A UniFrac-based principal coordinate analysis (PCoA) revealed that the bacterial composition of diarrheal piglets was significantly distinct from that of healthy piglets (*P* < 0.05, Fig. 2a). However, the α diversity of the fecal microbiota, as measured by the Chao index, did not show a significant difference between the two groups (*P* > 0.05, Fig. 2b). At the phylum level, Firmicutes and Bacteroidota were the two most abundant phyla in both healthy and diarrheal piglets (Fig. 2c). Diarrheal piglets had a higher relative abundance of Firmicutes and a lower relative abundance of Bacteroidota compared to healthy piglets (*P* < 0.05, Fig. 2d). Consequently, the Firmicutes to Bacteroidota ratio was significantly elevated in diarrheal piglets (*P* < 0.05, Fig. 2e).

At the genus level, diarrheal piglets had a significantly higher proportion of *Prevotella*, *Subdoligranulum*, *Collinsella*, *Blautia*, and *Ruminococcus gauvreauii*, and a significantly lower proportion of *Phascolarctobacterium* compared to healthy piglets (*P* < 0.05, Fig. 2f). At the species level, the relative abundances of *Lactobacillus amylovorus*, *Lactobacillus reuteri*, and *Ruthenibacterium lactatiformans* were significantly decreased, while the relative abundances of *Bacteroides sp.HF-5287* and *Bacteroides thetaiotaomicron* significantly increased in diarrheal piglets (*P* < 0.05, Fig. 2g). These results demonstrated that diarrheal piglets show gut microbial dysbiosis.

### Diarrheal piglets exhibit a decrease in fecal microbial production of tryptophan catabolites

**Fig. 3:**
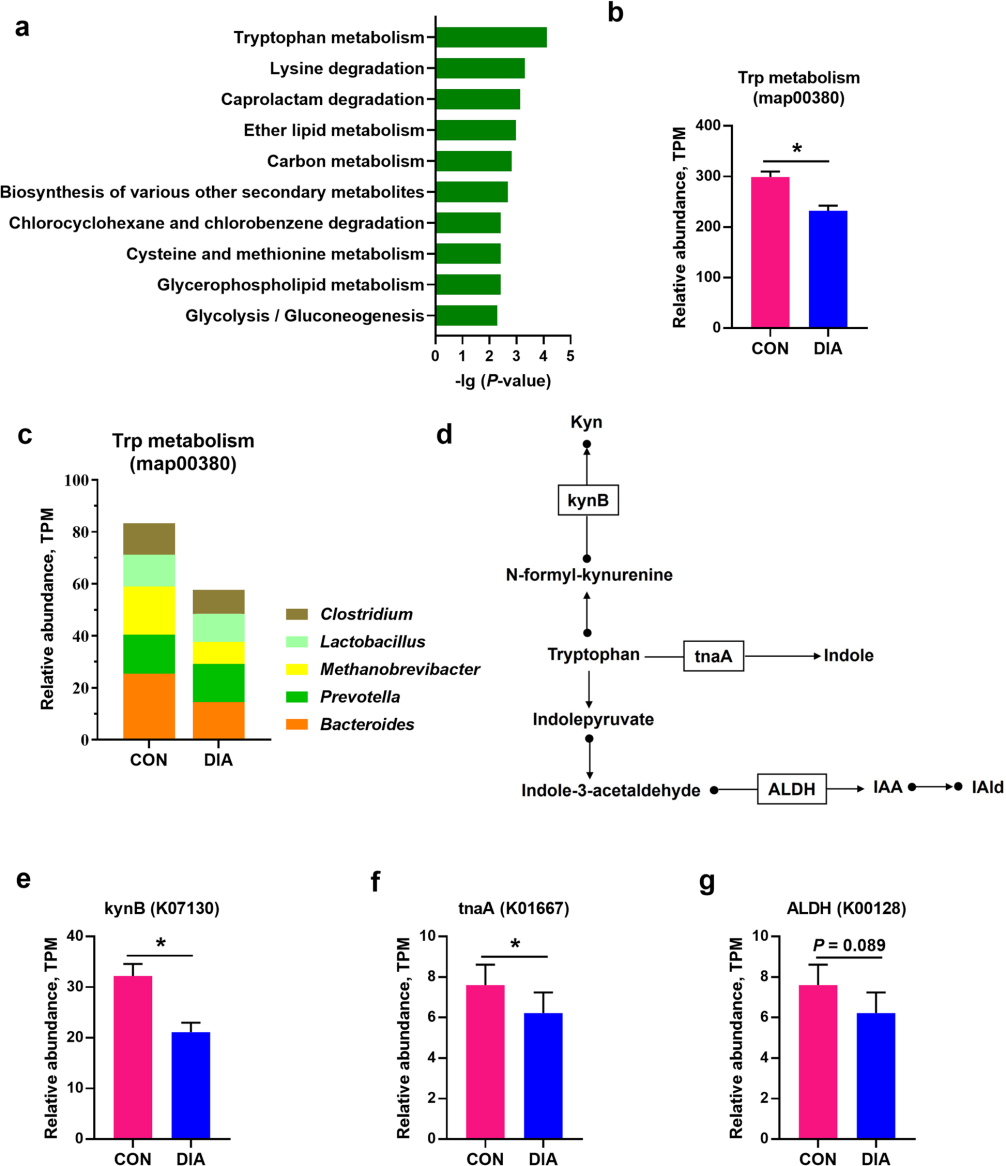
Differential functions of fecal microbiome regarding tryptophan metabolism between healthy and diarrheal weaned piglets. **a** Functional analysis of top 10 differential gene expression between CON and DIA groups based on *P* value. **b** The normalized relative abundance of DNA sequence reads mapping to the pathway of KEGG tryptophan metabolism (map00380). **c** The phylogenetic distributions of sequences in tryptophan metabolism at genus levels are based on the abundance of TPM assigned to each KO gene. **d** The cartoon displays the Trp catabolism to kynurenine (Kyn), indole, indole-3-acetic acid (IAA), and IAld and enzymes involved in each pathway. **e**–**g** The relative abundance of enzymes involved in the microbial catabolism of tryptophan in healthy and diarrheal piglets. KynB, kynurenine 3-monooxygenase. tnaA, tryptophanase. ALDH, aldehyde dehydrogenase. The results are expressed as the mean ± SEM (*n* = 10). ^*^P < 0.05

Next, we analyzed metagenomic sequencing data to investigate the differences in the functional profiles of the microbiome between healthy and diarrheal piglets. First, microbial KEGG pathways were compared. Tryptophan metabolism showed the most significant difference (*P* < 0.05, Fig. 3a). To assess the genetic potential of the microbiota to produce tryptophan metabolites, we determined the normalized relative abundance of DNA sequence reads mapped to the KEGG tryptophan metabolism pathway (map00380). The relative abundance of genes involved in tryptophan metabolism was significantly lower in diarrheal piglets compared to healthy piglets (*P* < 0.05, Fig. 3b). Further, the distributions of sequences in tryptophan metabolism at the genus level were analyzed based on the abundance of TPM assigned to each KEGG ortholog (KO) gene. Genes involved in tryptophan metabolism that had lower relative abundance in diarrheal piglets were mainly phylogenetically assigned to the genera *Bacteroides* (14.50 vs. 25.42), *Prevotella* (14.70 vs. 15.09), *Methanobrevibacter* (8.46 vs. 18.41), *Lactobacillus* (10.89 vs. 12.30), and *Clostridium* (9.12 vs. 12.02) (*P* < 0.05, Fig. 3c). We identified all known enzymes or KOs involved in microbial tryptophan catabolism in both healthy and diarrheal piglets. A total of 21 KOs were found, with 3 key KOs K07130 (*P* < 0.05), K01667 (*P* < 0.05), and K00128 (0.05 < *P *< 0.10) showing changes or a trend of change between healthy and diarrheal piglets (Fig. 3d–g). The relative abundances of genes encoding the enzymes kynurenine 3-monooxygenase (kynB), tryptophanase (tnaA), and aldehyde dehydrogenase (ALDH), were responsible for converting tryptophan to kynurenine (Kyn), indole, indole-3-acetic acid (IAA), or IAld (Fig. 3d). The enzymes kynB (*P* < 0.05) and tnaA (*P* < 0.05) were significantly lower, and ALDH (0.05 < *P* < 0.10) showed a decreasing trend in diarrheal piglets compared to healthy piglets (Fig. 3d–g). This suggested that piglets with diarrhea exhibit a significant downregulation in the genetic potential of their microbiota to produce tryptophan metabolites.

**Fig. 4:**
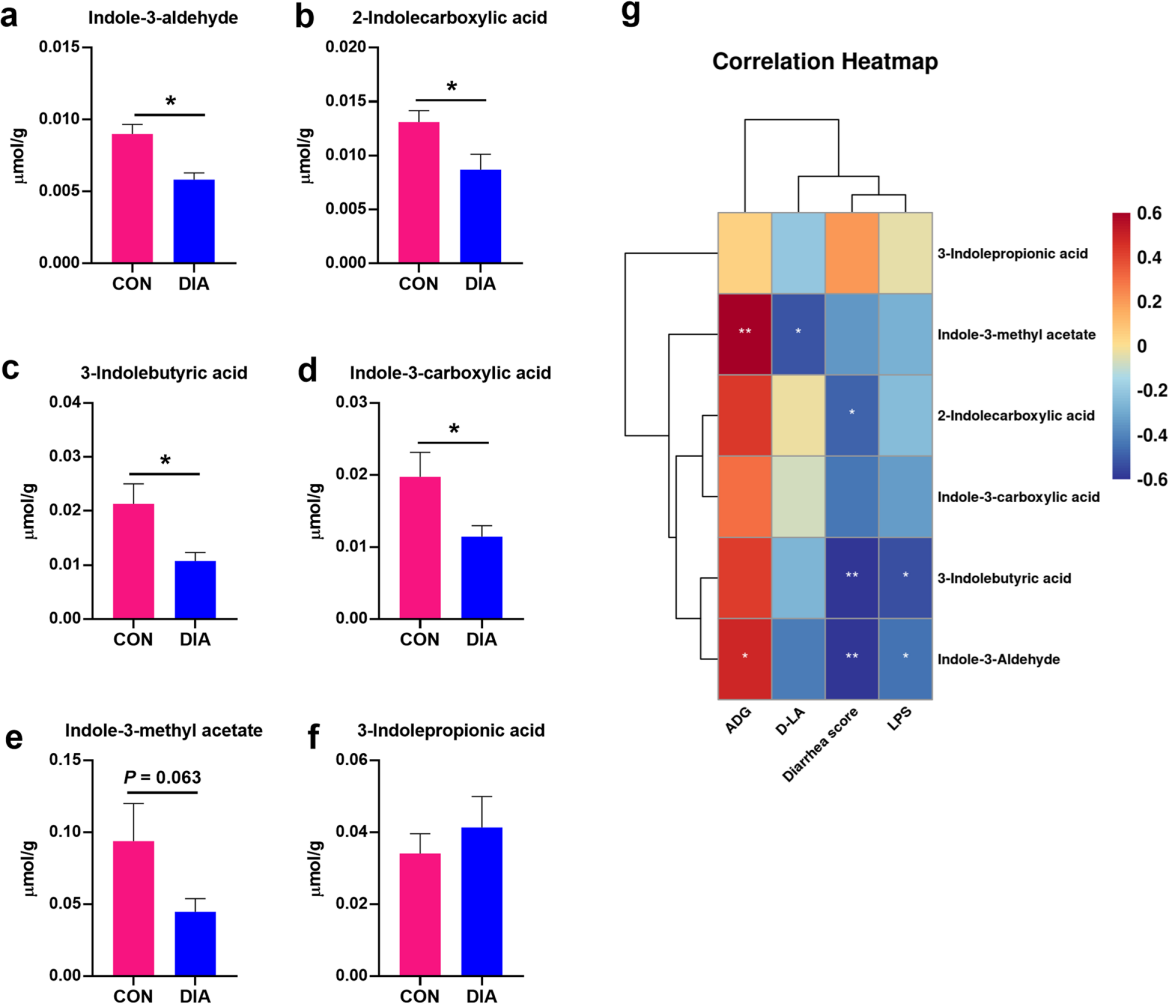
The difference in microbial production of tryptophan metabolites between healthy and diarrheal weaned piglets and correlations between tryptophan metabolites and diarrhea-related indicators. **a**–**f** The concentration of IAld, 2-indolecarboxylic acid, 3-indolebutyric acid, indole-3-carboxylic acid, indole-3-methyl acetate, and 3-indolepropionic acid in the feces of piglets. **g** The correlation analysis between the six tryptophan catabolites and diarrhea-related indicators. Data are expressed as the mean ± SEM (*n* = 10). ^*^P < 0.05

To further explore the effects of diarrhea on the microbial production of tryptophan metabolites, we used targeted metabolomics to detect the concentrations of key tryptophan metabolites in the feces of healthy and diarrheal piglets. Six tryptophan catabolites were found and four catabolites, including IAld, 2-indolecarboxylic acid, 3-indolebutyric acid, indole-3-carboxylic acid, showed significant decreases in diarrheal piglets compared to healthy piglets (*P* < 0.05, Fig. 4a–d). Indole-3-methyl acetate exhibited a decreasing trend in diarrheal piglets compared to healthy piglets (0.05 < *P* < 0.10, Fig. 4e). Additionally, we identified correlations between these six tryptophan catabolites and diarrhea-related indicators. The diarrhea score and serum LPS concentration were negatively correlated with most of the tryptophan catabolites, including IAld, 3-indolebutyric acid, and 2-indolecarboxylic acid (*P* < 0.05, Fig. 4g). Serum D-LA concentration was negatively correlated with indole-3-methyl acetate (*P* < 0.05, Fig. 4g). Conversely, the ADG was positively correlated with the concentrations of IAld and indole-3-methyl acetate in the feces of the piglets. Collectively, these data demonstrated that diarrheal piglets exhibited a decreased microbial production of tryptophan metabolites.

### Indole-3-aldehyde promotes gut barrier function and intestinal epithelial cell proliferation in the small intestine of weaned piglets

**Fig. 5:**
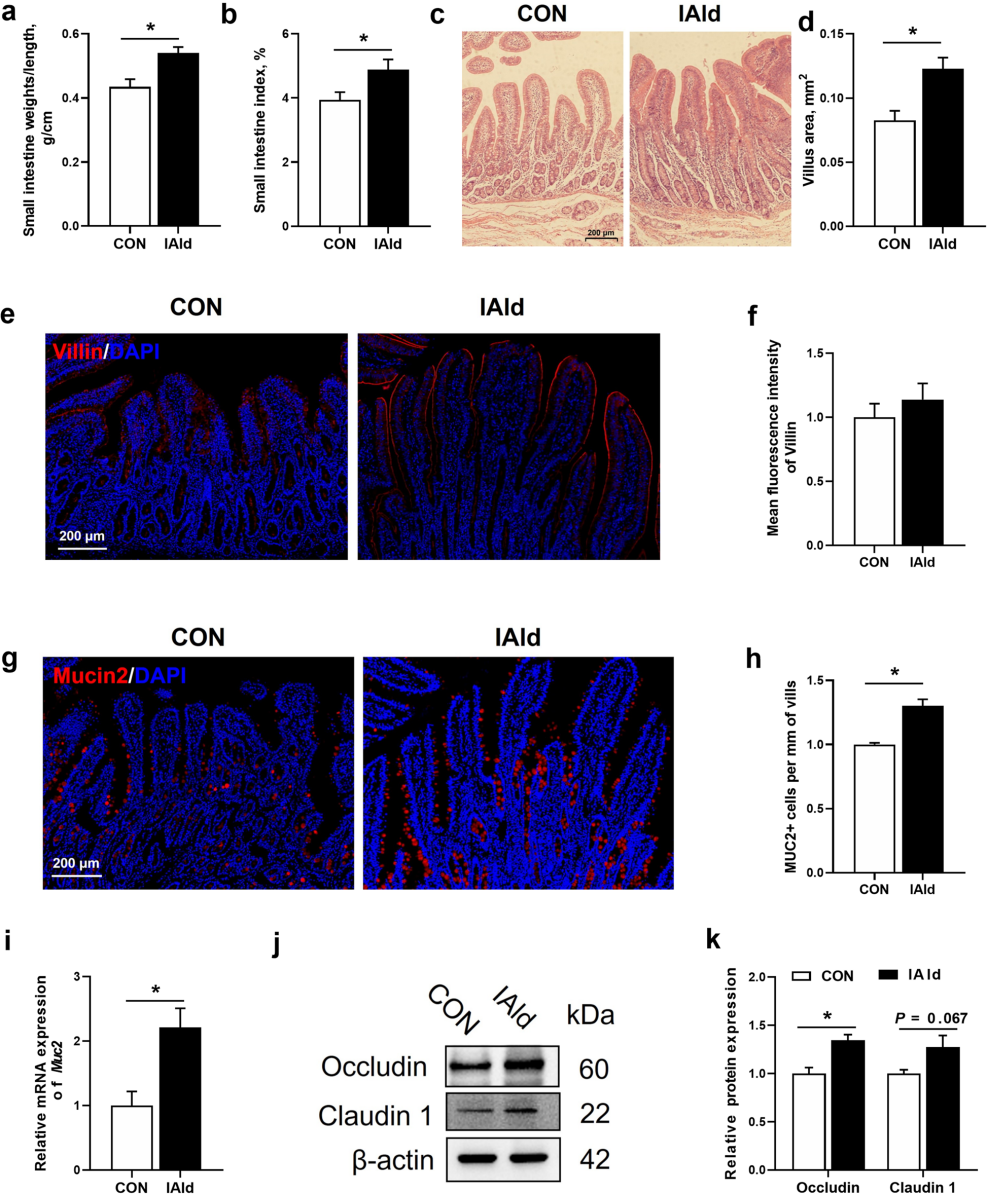
The effects of IAld on histology and gut barrier function in the small intestine of weaned piglets. **a** The ratio of small intestinal weight to length. **b **The small intestinal index. **c** Representative H&E staining of small intestinal tissue sections from the CON and IAld groups of piglets (Scale bars, 200 μm). **d** The villus area. **e** Representative images of CON and IAld groups of piglets immunostained with an anti-Villin antibody (red) and counterstained with DAPI (blue) (Scale bars, 200 μm) in the small intestine. **f** Mean fluorescence intensity of Villin in the small intestine. **g** Representative images of CON and IAld groups of piglets immunostained with an anti-Mucin2 antibody (red) and counterstained with DAPI (blue) (Scale bars, 200 μm) in the small intestine. **h** Mucin2-positive cell per villi of the small intestine. **i** Relative mRNA expression of *MUC2*. **j** Immunoblot analysis of Occludin and Claudin1 in the small intestine (*n* = 4). **k** Relative protein expression levels of Ocludin and Claudin1 in the small intestine (*n* = 4). Data are expressed as the mean ± SEM. (a–i) *n* = 8. ^*^P < 0.05

To investigate the role of IAld, a key tryptophan metabolite from the microbiota, in the intestinal barrier function and development of weaned piglets, we conducted a two-week dietary intervention by supplementing the basic diet with 100 mg/kg IAld. Following the intervention, the small intestinal weight/length ratio and the small intestinal index were significantly increased in the IAld group compared to the CON group of piglets (*P* < 0.05, Fig. 5a and b). Dietary IAld supplementation also significantly improved small intestine morphology by increasing the villus area (*P* < 0.05, Fig. 5c and d). To further explore the influence of IAld on gut barrier function, we performed immunofluorescence staining for Villin and Mucin2. Notably, dietary supplementation with 100 mg/kg IAld significantly increased the number of goblet cells (marked by Mucin2) and the mRNA expression of *Muc2* (*P* < 0.05, Fig. 5g–i). In contrast, the number of enterocytes (marked by Villin) in the small intestine did not significantly differ between the IAld and CON groups (*P* > 0.05, Fig. 5e and f). Furthermore, the expression of the tight junction proteins Occludin was upregulated (*P* < 0.05), while Claudin 1 expression showed a tendency to increase (0.05 < *P* < 0.10) in the IAld group (Fig. 5j and k).

**Fig. 6:**
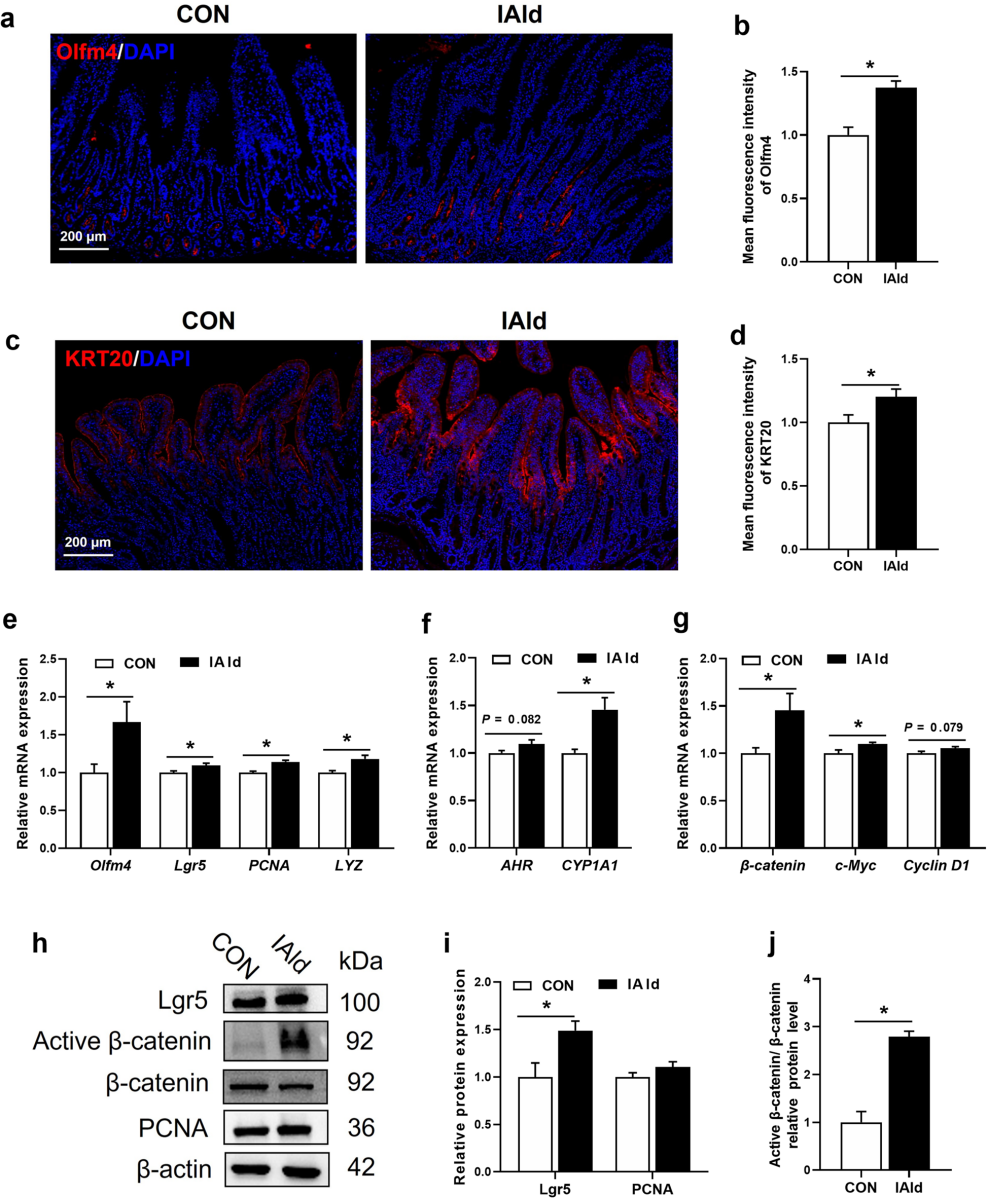
The effects of IAld on intestinal epithelial cell proliferation and differentiation in the small intestine of weaned piglets. **a** Representative images of CON and IAld groups of piglets immunostained with an anti-Olfm4 antibody (red) and counterstained with DAPI (blue) (Scale bars, 200 μm) in the small intestine. **b** Mean fluorescence intensity of Olfm4 in the small intestine. **c** Representative images of CON and IAld groups of piglets immunostained with an anti-KRT20 antibody (red) and counterstained with DAPI (blue) (Scale bars, 200 μm) in the small intestine. **d** Mean fluorescence intensity of KRT20 in the small intestine. **e** The relative mRNA expression of *Olfm4*, *Lgr5*, *PCNA*, and *LYZ* in the small intestine. **f** The relative mRNA expression of *AHR* and *CYP1A1* in the small intestine. **g** The relative mRNA expression of *β-catenin*, *c-Myc*, and *Cyclin D1* in the small intestine. **h** Immunoblot analysis of Lgr5, active β-catenin, β-catenin, and PCNA in the small intestine (*n* = 4). **i** Relative protein expression levels of Lgr5 and PCNA in the small intestine. **j** The ratio of the relative protein expression levels of active β-catenin to β-catenin in the small intestine. Data are expressed as the mean ± SEM. (a–g) *n* = 8. ^*^P < 0.05

Next, we explored the impacts of IAld on the proliferation and differentiation of intestinal epithelial cells. Immunofluorescence staining revealed that IAld supplementation significantly increased the number of Olfm4^+^ ISCs (*P* < 0.05, Fig. 6a and b) and significantly promoted ISC differentiation (marked by KRT20) in the small intestine (*P* < 0.05, Fig. 6c and d). Additionally, the mRNA expression levels of makers of ISCs (*Olfm4* and *Lgr5*), proliferation (*PCNA*), and Paneth cells (*LYZ*) were upregulated in the IAld group (*P* < 0.05, Fig. 6e). IAld supplementation upregulated the mRNA expression of *CYP1A1* (an AHR target gene), *β-catenin*, and the Wnt/β-catenin target gene *c-Myc* (*P* < 0.05, Fig. 6f and g). The mRNA expression of *AHR* (an IAld ligand) and the Wnt/β-catenin target gene *Cyclin D1* exhibited an increasing trend in IAld group (0.05 < *P* < 0.10, Fig. 6f and g). The protein expression of Lgr5 and active β-catenin/β-catenin were also upregulated in the IAld group (*P* < 0.05, Fig. 6h–j). Taken together, these findings suggested that IAld plays a vital role in promoting gut barrier function and ISC expansion in the small intestines of weaned piglets.

### Indole-3-aldehyde promotes intestinal stem cell expansion

**Fig. 7:**
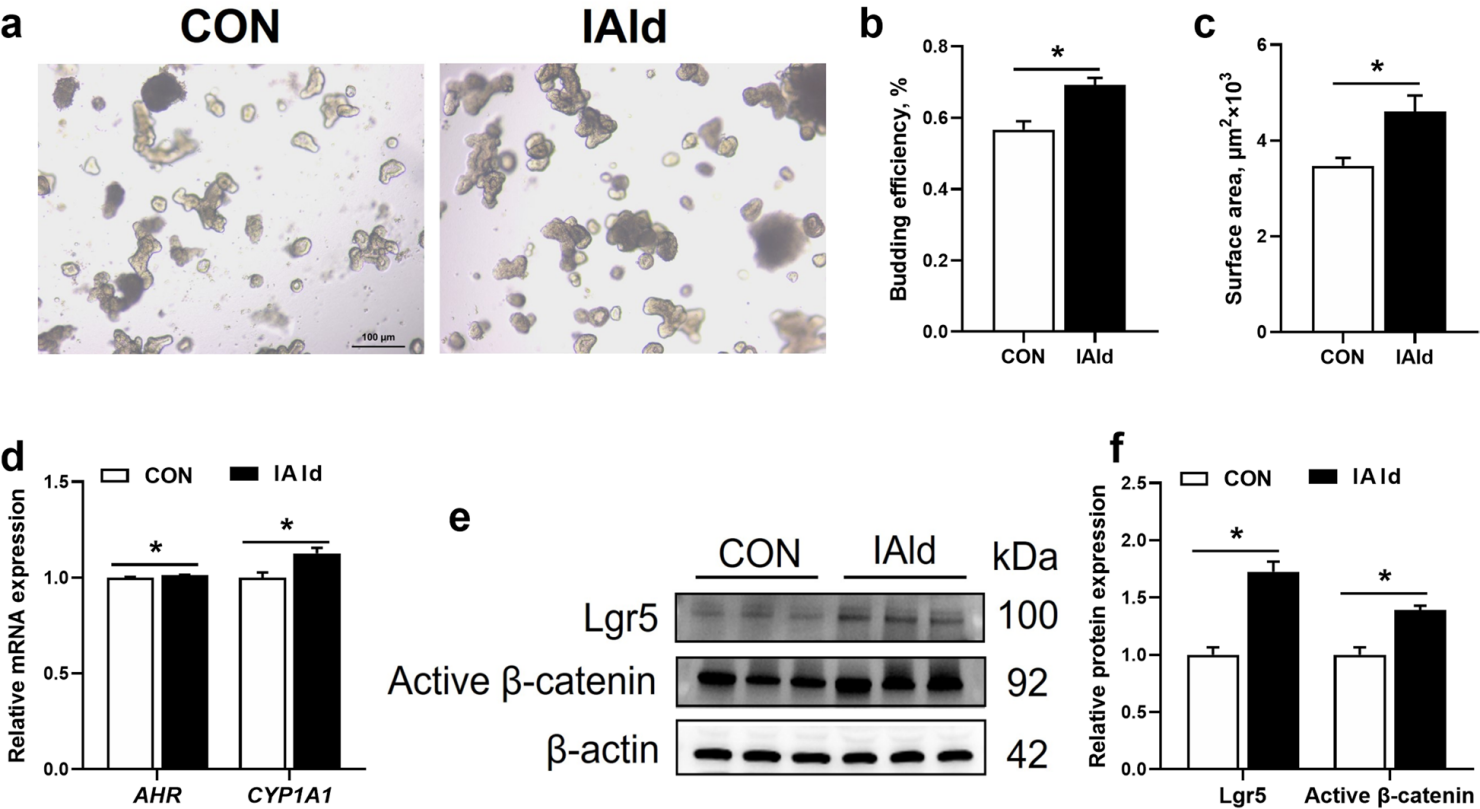
The effects of IAld on porcine organoid proliferation in the small intestine. **a** Representative images of the intestinal organoids on day 3 (Scale bars, 100 μm). **b** and **c** The budding efficiency and surface area of the organoid in pigs. **d** The relative mRNA expression of *AHR* and *CYP1A1* in the organoid of pigs. **e** Immunoblot analysis of Lgr5 and active β-catenin in the organoid of pigs. **f** Relative protein expression levels of Lgr5 and active β-catenin in the organoid of pigs. Data are expressed as the mean ± SEM (*n* = 3 well per group).^*^P < 0.05

Organoids grown from isolated intestinal crypts are commonly used as models to recapitulate the normal physiology of the intestine [27]. To further confirm the role of IAld in promoting ISC expansion, we utilized a small intestinal organoid model in pigs. IAld significantly enhanced the budding efficiency and surface area of the organoids (*P* < 0.05, Fig. 7a–c). Additionally, mRNA expression levels of *AHR* and *CYP1A1* were significantly elevated in the IAld group (*P* < 0.05, Fig. 7d). The protein expression levels of Lgr5 and active β-catenin were also significantly higher in the IAld group compared to the control group (*P* < 0.05, Fig. 7e and f). Collectively, these findings indicated that IAld promotes ISC proliferation in the small intestine of piglets.

## Discussion

It is widely recognized that post-weaning diarrhea is closely linked to gut microbiota dysbiosis. To identify the key gut microbial species and microbial metabolites in the antidiarrhea process of weaned piglets, we first compared the intestinal microbiota and their metabolites between diarrheal and healthy piglets. The results revealed that tryptophan catabolites produced by intestinal microbiota were significantly reduced in piglets suffering from diarrhea. Further, we demonstrated that IAld, a crucial tryptophan catabolite, improved intestinal development and barrier function by promoting ISC expansion in piglets.

Post-weaning diarrhea is typically associated with disturbances in the intestinal barrier, characterized by increased intestinal permeability [5, 28]. As shown in our study, elevated serum levels of D-LA and LPS in diarrheal piglets suggested increased intestinal permeability, leading to the translocation of luminal bacteria, toxins, and antigens into subepithelial tissues. This translocation may be a crucial cause of post-weaning diarrhea [29].

Gut microbiota dysbiosis is a hallmark of post-weaning diarrhea [30]. The microbiota analysis in this study revealed that weaning-diarrhea changed the structure and composition of the gut microbiota in piglets. The ratio of Firmicutes to Bacteroidota is widely accepted as playing an essential role in maintaining intestinal homeostasis and is often used as an indicator of inflammatory bowel disease (IBD) [31, 32]. In the present study, diarrheal piglets exhibited a higher ratio of Firmicutes to Bacteroidota, suggesting a decreased capacity of the microbiome to maintain intestinal homeostasis in these piglets. Numerous studies have supported the concept that microbiota dysbiosis is associated with a reduction in probiotics and an increase in enteric pathogens. For example, Li et al. reported that gut microbiota dysbiosis in diarrheal piglets was characterized by a deficiency in *Lactobacillus*, an abundance of *Escherichia coli*, and enriched lipopolysaccharide biosynthesis [33]. Notably, we found a decreased abundance of *Lactobacillus amylovorus* and *Lactobacillus reuteri*, along with an increased abundance of *Bacteroides sp.HF-5287* and *Bacteroides thetaiotaomicron* in diarrheal piglets. *Lactobacilli *spp*.* is widely recognized as beneficial probiotics that improve intestinal barrier function [33, 34]. *Bacteroides thetaiotaomicron* was reported to shift its metabolism from dietary polysaccharides to host-derived mucus glycans when dietary polysaccharides are scarce, ultimately leading to the destruction of the mucus barrier [35]. Importantly, these microbial changes were accompanied by an increase in LPS production in diarrheal piglets, which can have a detrimental impact on the gut epithelial lining [36]. Overall, these findings revealed that piglets with diarrhea exhibited a disordered microbiome characterized by significant shifts in microbial composition and function, which are linked to adverse health outcomes.

Metabolites produced by the gut microbiota are recognized as key signaling molecules in host-microbial cross-talk [37]. In this study, the functional profiles of the microbiome showed that among various metabolic pathways, tryptophan metabolism showed the most significant difference between healthy piglets and those suffering from diarrhea. Specifically, the potential for microbiota to produce tryptophan catabolites was notably reduced in diarrheal piglets compared to healthy piglets. In the gut microbiota of piglets, tryptophanases were widely expressed in *Bacteroides* spp., *Prevotella* spp., *Methanobrevibacter* spp., *Lactobacillus* spp., and *Clostridium* spp. Some of them, including *Lactobacillus* spp., *Bacteroides* spp., and *Clostridium* spp., have been reported to metabolize tryptophan [21, 38]. Notably, our data showed that *Bacteroides* spp. is the dominant genus producing tryptophanases and tryptophan metabolites. Several *Bacteroides* species have been reported to produce indole, IAA, indole-3-lactic acid (ILA) [38, 39]. We further identified the key enzymes involved in tryptophan metabolism, including KynB, tnaA, and ALDH, which showed lower abundance in piglets with diarrhea. The enzyme tnaA, which is expressed in both gram-negative and gram-positive bacteria, can convert tryptophan into indole [38, 40]. The KynB enzyme is responsible for the biosynthesis of kynurenine (Kyn), while ALDH participates in the synthesis of IAA and IAld [41, 42]. These identified enzymes further support the notion that tryptophan metabolites can be produced by gut microbiota. Based on the above research, we speculated that bacteria, such as *Bacteroides* spp. and *Clostridium* spp., may collectively contribute to a decrease in IAA or IAld in diarrheal piglets.

Accumulating studies have revealed that microbial tryptophan metabolites play a pleiotropic role in regulating various physiological processes [43, 44]. For example, microbe-derived IAld was reported to protect against Candidiasis and Colitis by promoting interleukin-22 production [21]. In the present study, several tryptophan catabolites, including IAld, 2-indole carboxylic acid (2-ICA), 3-indolebutyric acid (IBA), indole-3-carboxylic acid (I3CA), and indole-3-methyl acetate, were present at lower levels in diarrheal piglets. A correlation analysis between the six tryptophan catabolites and diarrhea-related indicators demonstrated that the levels of the tryptophan metabolites, including IBA and IAld, were negatively associated with the diarrhea score and serum LPS levels. These findings suggested that changes in tryptophan metabolites, especially IAld, are associated with the incidence of diarrhea in piglets.

Indole-3-aldehyde is a well-known tryptophan metabolite produced by gut microbiota [44]. To investigate the role of tryptophan metabolites in the host’s physiological processes, IAld was selected for dietary supplementation in weaned piglets. Intestinal barrier function relies on intricate crosstalk among the gut microbiota, mucus layer, tight junction proteins, and the intestinal epithelium [45]. We hypothesize that microbiota-derived IAld plays a crucial role in the development of intestinal epithelium and even the function of the intestinal barrier. As shown in the study, dietary IAld improved villous morphology, increased the number of MUC2^+^ cells, and enhanced the expression of Villin and the tight junction proteins in weaned piglets. The mucin MUC2 is the main protein component of the mucus layer and plays a vital role in protecting the intestinal epithelium [46]. Villin, a major microfilament-associated protein located in the brush border microvilli, is an essential marker for the development of the intestinal epithelium [47]. Thus, the results demonstrated that dietary IAld promoted mucosal barrier function in the small intestine of weaned piglets.

The development of the intestinal epithelium during the weaning period profoundly affects the long-term phenotype and intestinal barrier function of pigs. Intestinal stem cells, particularly the Lgr5^+^ crypt-base columnar cells, drive the constant development of the intestinal epithelium [48]. We found that dietary IAld significantly promoted intestinal epithelial cell proliferation. Notably, IAld promoted Lgr5^+^ ISC proliferation in vivo and ex vivo. The canonical Wnt/β-catenin pathway is crucial for determining the fate of ISCs [49, 50]. In this study, IAld activated the Wnt/β-catenin signaling, modulating the expression of downstream target genes such as *c-Myc* and *Cyclin D1*, while upregulating the mRNA expression of *LYZ*, a biomarker for Paneth cells. In addition, AHR activation has been shown to regulate intestinal epithelium development in a ligand-specific manner [51, 52]. Interestingly, we found that dietary IAld upregulated the expression of AHR (an IAld ligand) and CYP1A1 (an AHR target gene), suggesting that IAld activates the AHR signaling pathway. Similarly, indoleacrylic acid, another microbial tryptophan metabolite, exhibited anti-inflammatory effects by activating AHR in mice [21]. In addition, recent studies have indicated that the Wnt/β-catenin pathway can be activated by AHR [53, 54]. The molecular mechanism by which IAld-AHR signaling regulates the Wnt/β-catenin pathway and thus affects ISC expansion needs further study. Taken together, dietary IAld promoted ISC expansion in the small intestine of weaned piglets.

## Conclusions

**Fig. 8:**
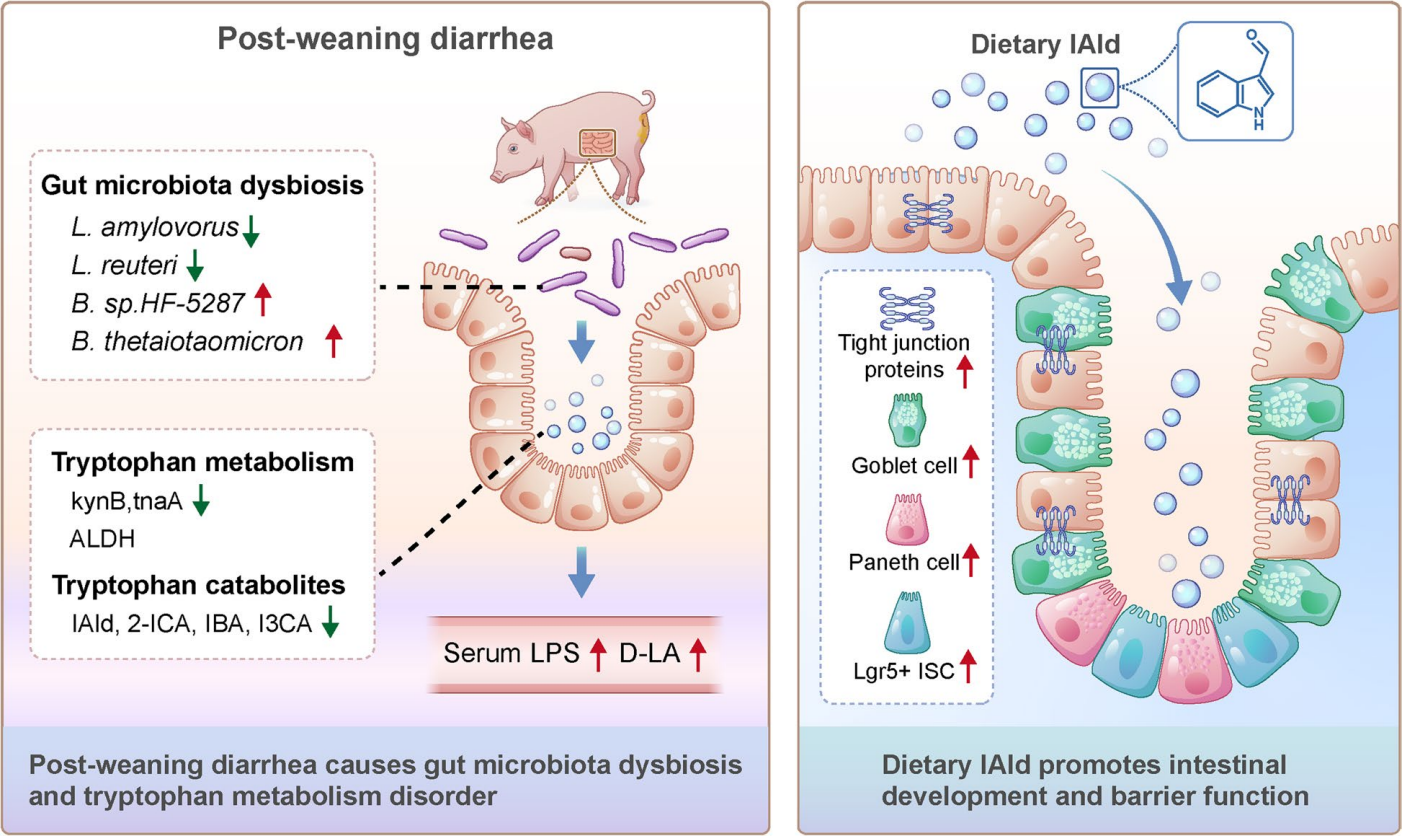
Post-weaning diarrhea causes gut microbiota dysbiosis, and consequent tryptophan metabolism disorder, leading to an increase in the serum LPS and D-LA levels. Dietary IAld, a microbiota-derived tryptophan metabolite, improves intestinal development and barrier function by promoting ISC expansion in weaned piglets. IAld, indole-3-aldehyde; 2-ICA, 2-indolecarboxylic acid; IBA, 3-indolebutyric acid; I3CA, indole-3-carboxylic acid; LPS, lipopolysaccharide; D-LA, D-Lactate; ISC, intestinal stem cell

In summary, we demonstrated that post-weaning diarrhea causes gut microbiota dysbiosis, and consequent tryptophan metabolism disorder, leading to an increase in the serum LPS and D-LA levels. Further, we revealed that dietary IAld, a key microbial tryptophan metabolite, improves intestinal development and barrier function by promoting ISC expansion in weaned piglets (Fig. 8).

## Data Availability

The datasets used during the current study are available from the corresponding author on reasonable request.

## Abbreviations

ADG: Average daily gain
AHR: Aryl hydrocarbon receptor
ALDH: Aldehyde dehydrogenase
D-LA: D-Lactate
IAA: Indole-3-acetic acid
IAld: Indole-3-aldehyde
KRT20: Keratin 20
kynB: kynurenine 3-monooxygenase
Lgr5: Leucine-rich repeat-containing G-protein coupled receptor 5
LPS: Lipopolysaccharide
LYZ: Lysozyme
MUC2: Mucin2
Olfm4: Olfactomedin 4
PCNA: Proliferating cell nuclear antigen
tnaA: Tryptophanase
